# Potential diagnostic of lymph node metastasis and prognostic values of TM4SFs in papillary thyroid carcinoma patients

**DOI:** 10.3389/fcell.2022.1001954

**Published:** 2022-12-08

**Authors:** Kun Wang, Haomin Li, Junyu Zhao, Jinming Yao, Yiran Lu, Jianjun Dong, Jie Bai, Lin Liao

**Affiliations:** ^1^ Department of Endocrinology and Metabology, Liaocheng People’s Hospital, Liaocheng, Shandong, China; ^2^ Department of Endocrinology and Metabology, Shandong Qianfoshan Hospital, Cheeloo College of Medicine, Shandong University, Jinan, Shandong, China; ^3^ Department of Endocrinology and Metabology, The First Affiliated Hospital of Shandong First Medical University & Shandong Provincial Qianfoshan Hospital, Jinan, Shandong, China; ^4^ Department of Endocrinology, Qilu Hospital of Shandong University, Jinan, Shandong, China

**Keywords:** papillary thyroid carcinoma, lymph node metastasis, TM4SFs, prognostic, diagnostic

## Abstract

**Background:** Although the prognosis of papillary thyroid carcinoma (PTC) is relatively good, it causes around 41,000 deaths per year, which is likely related to recurrence and metastasis. Lymph node metastasis (LNM) is an important indicator of PTC recurrence and transmembrane 4 superfamily (TM4SF) proteins regulate metastasis by modulating cell adhesion, migration, tissue differentiation, and tumor invasion. However, the diagnostic and prognostic values of TM4SF in PTC remain unclear.

**Methods:** This study aimed to identify TM4SF genes with predictive value for LNM and prognostic value in PTC using bioinformatic analysis. We screened the differentially expressed genes (DEGs) of the TM4SF family in PTC using data from TCGA, constructed a PPI network using STRING, and evaluated the predictive role of *TM4SF1* in LNM *via* a binary logistic regression analysis and ROC curve. We assessed the association between *TM4SF1* expression and DNA methylation, and determined the functional and mechanistic role of *TM4SF1* in promoting LNM *via* GSEA, KEGG, and GO. We estimated the relationship between each TM4SF gene and overall survival (OS, estimated by Kaplan-Meier analysis) in patients with PTC and established a predictive model of prognostic indicators using a LASSO penalized Cox analysis to identify hub genes. Finally, we explored the correlation between TM4SFs and TMB/MSI.

**Results:** We identified 21 DEGs from the 41 TM4SFs between N0 (without LNM) and N1 (with LNM) patients, with *TM4SF1*, *TM4SF4*, *UPK1B*, and *CD151* being highly expressed in the N1 group; several DEGs were observed in the TNM, T, and N cancer stages. The “integrins and other cell-surface receptors” pathway was the most significantly enriched functional category related to LNM and TM4SFs. *TM4SF1* was identified as an indicator of LNM (AUC= 0.702). High levels of *TM4SF1* might be related to Wnt/β-catenin pathway and epithelial–mesenchymal transition (EMT) process in PTC. The higher expression of *TM4SF1* was also related to DNA promoter hypomethylation. *CD9*, *TM4SF4*, *TSPAN2*, and *TSPAN16* were associated with OS in PTC patients and TSPAN2 has great potential to become a prognostic marker of PTC progression. For the prognostic model, the riskscore = (-0.0058)*CD82+(-0.4994)*+(0.1584)*TSPAN11+(1.7597)*TSPAN19+(0.2694)*TSPAN2 (lambda.min = 0.0149). The AUCs for 3-year, 5-year, and 10-year OS were 0.81, 0.851, and 0.804. *TSPAN18*, *TSPAN31*, and *TSPAN32* were associated with both TMB and MSI in PTC patients.

**Conclusion:** Our findings identified *TM4SF1* as a potential diagnostic marker of LNM and *TSPAN2* as a prognostic factor for patients with PTC. Our study provides a novel strategy to assess prognosis and predict effective treatments in PTC.

## 1 Introduction

THCAs can be divided into different histological types: papillary, follicular, Hürthle cell, anaplastic, and medullary thyroid carcinoma (Laetitia et al., 2020). Papillary thyroid carcinoma (PTC) is the most common subtype of thyroid carcinoma (THCA), accounting for about 80%–90% of all case (Laetitia et al., 2020). PTC causes ∼41,000 deaths per year (Cabanillas et al., 2016) with about 20% of patients with early PTC experiencing recurrence and metastasis after surgery, and recurrent PTC can develop into undifferentiated THCA with extremely poor prognosis (Ibrahim and Busaidy, 2017; Sapuppo et al., 2019). The recurrence and/or metastasis after resistance to radioiodine (RAI) treatment can affect between 5% and 15% of patients with differentiated thyroid cancer (DTC) and seriously impact the prognosis. Nearly 70% of these patients will become RAI-refractory (RR-DTC) and the average life expectancy will be significantly shortened by 3–5 years (Van Nostrand, 2018; Fugazzola et al., 2019). Prophylactic neck dissection has been a common practice to determine lymph node metastasis (LNM) and, consequently, PTC recurrence (Patron et al., 2012), but recent studies have shown that only 64% of patients that experience recurrence present with LNM (Kluijfhout et al., 2017). Therefore, an urgent need exists for reliable biomarkers to predict the occurrence of LNM and prognosis of PTC and avoid diagnostic operations that promote postoperative complications and reduce quality of life.

The transmembrane 4 superfamily (TM4SF), or tetraspanins, are a large family of evolutionarily conserved proteins with four transmembrane domains. Which occur in eukaryotic cell membranes. The gene family includes 33 classical genes (*TSPAN1–TSPAN33*) (Todres et al., 2000) and 8 newly described tetraspanin genes (*TM4SF1, −4, −5, −10, −11, −18, −19, −20*) (*Wright et al., 2000*). Their localization and glycosylation on the cell membrane indicate that they participate in the interaction between cells and the extracellular matrix, determining cell adhesion, migration, tissue differentiation, tumor invasion, and metastasis, among other cell behaviors (Romanska and Berditchevski, 2011). Increasing evidence suggests that these proteins are biomarkers of some advanced malignancies, such as gastric carcinoma, THCA, and cervical cancer (Chen et al., 2011; Romanska and Berditchevski, 2011; Holters et al., 2013). As of yet, it is unclear what role TM4SF proteins play in the pathogenesis and prognosis of PTC.

Through bioinformatic analysis, we sought to determine the predictive and prognostic values of TM4SF genes in LNM and PTC.

## 2 Materials and methods

### 2.1 Data collection and microarray data

The Cancer Genome Atlas (TCGA) (https://tcga-data.nci.nih.gov/tcg) database was used to download the RNA-sequencing expression profiles (level 3) and clinical records data of 1,106 THCA patients (Blum et al., 2018). THCA data included 711 patients without (“normal” group) and 395 with PTC (“tumor” group; 163 without LNM [N0] and 206 with LNM [N1]).

### 2.2 Differentially expressed transmembrane 4 superfamily genes

According to the median levels of mRNA expression, patients with PTC were divided into high and low expression groups. The ggplot2 (v3.3.3) package in R software (v4.0.3) was used to identify differentially expressed genes (DEGs) between the normal vs tumor and N0 vs N1 groups (Chen et al., 2004).

Funrich software (Pathan et al., 2017) (http://www.funrich.org/) was used to verify the occurrence of the same genes in two or three datasets.

### 2.3 Binary logistic regression analysis and receiver operating characteristic curve

Binary logistic regression analysis was used to estimate independent factors. Receiver operating characteristic (ROC) curve analysis, estimating the area under the ROC curve (AUC), was used to screen genes with the highest predictive values for LNM. The best sensitivity/specificity relationship was determined using a cut-off point extrapolated from the AUC. Both logistic regression and ROC curve analyses were conducted in SPSS v25.0 (SPSS IBM, Chicago, IL, United States).

### 2.4 Analyses of *TM4SF1* in different thyroid cancer cell lines

A cell line mRNA expression matrix of THCA tumors was obtained from the CCLE database (Ghandi et al., 2019), and *TM4SF1* expression was analyzed using the ggplot2 package in R software.

### 2.5 Methylation analysis of *TM4SF1*

Survival Meth—a webserver of cancer-associated methylation, based on the TCGA, CCLE, and GEO databases (Zhang et al., 2021)was used to investigate the effect of *TM4SF1* DNA methylation on protein expression and THCA prognosis. Survival Meth calculated the risk score of each.

Sample based on the formula: Risk score = *β*1x1 + *β*2x2 +···+ βi x xi, where xi was the methylation value of DMFE or the clinical data and βi was its corresponding regression coefficient obtained above. The higher risk score was, the poorer prognosis patient was.

Shiny Methylation Analysis Resource Tool (SMART) (http://www.bioinfo-zs.com/smartapp) (Li et al., 2019) was used to analyze the levels of *TM4SF1* DNA methylation in THCA patients.

### 2.6 Protein–protein interaction and co-expression analyses of transmembrane 4 superfamily

Search Tool for the Retrieval of Interacting Genes (STRING) (https://string-db.org/)database was used to construct the protein–protein interaction (PPIs) network of the DEGs (Szklarczyk et al., 2021).

GEPIA database (http://gepia.cancer-pku.cn/) (Tang et al., 2017) and the ClusterProfiler package in R (Yu et al., 2012) was used to determine co-expression genes of TM4SF family in THCA *via* RNA sequencing data.

### 2.7 KEGG pathway, GO enrichment, and GSEA of transmembrane 4 superfamily

KEGG (www.kegg.jp/kegg/kegg1.html) (Kanehisa et al., 2021) and DAVID (Dennis et al., 2003) databases were used to perform KEGG and GO function enrichment analyses. The GO analysis considered the biological process, cellular component, and molecular function, with a selection criterion of DEGs >2 per term, and statistical significance at *p* < 0.05.

### 2.8 Survival analysis and immunoassay of transmembrane 4 superfamily

TCGA-THCA was used to analyze the relationship between TM4SFs mRNA expression and overall survival (OS, estimate) in PTC patients by Kaplan-Meier analysis.

For Kaplan-Meier curves, *p*-values and hazard ratio (HR) with 95% confidence interval (CI) were generated by log-rank tests and univariate Cox proportional hazards regression. The KM survival analysis with log-rank test were also used to compare the survival difference between above two groups. Then we performed multivariate COX analysis survival analysis to screen the independent influencing factors of survival. All the analysis methods and R packages were implemented by R (foundation for statistical computing 2020) version 4.0.3. *p* value < 0.05 was considered statistically significant.

The “SCNA”, “Gene”, and “Survival” module of the Tumor Immune Estimation Resource (TIMER) database (https://cistrome.shinyapps.io/timer/) (Li et al., 2017) were used to estimate the levels of tumor-infiltrating immune cells (TIICs) associated with TM4SFs based on somatic copy number alterations (SCNAs), the association between gene expression, tumor purity, and immune infiltration levels and the survival curves associated with high and low levels of tumor infiltrating immune cells. A Wilcoxon rank-sum test was used to compare infiltration levels for each SCNA in PTC with that in normal tissues.

### 2.9 Construction of the prognostic model

SPSS 26.0 software (IBM Germany GmbH, Ehningen, Germany) was used to assessment whether there was a multiple collinearity relationship among genes. Collinearity diagnostics was performed by “Multiple linear regression” module, VIF> 5 was considered to have multicollinearity, and VIF> 10 was considered to have serious multicollinearity.

To eliminate the influence of multicollinearity on regression analysis, LASSO-penalized Cox regression analysis was adopted to identify hub genes and constructed the prognostic model. The best penalty parameter is estimated through 10 times cross validation in the training data set (Tibshirani, 1997). Prognostic gene marker expressed as risk score=(coefficient mRNA1×mRNA expression)+(coefficient mRNA 2×mRNA2 expression)+......+(mRNAn coefficient×mRNAn expression). Taking the median risk score as the threshold, 397 patients were divided into high-risk group and low-risk group. Kaplan Meier (KM) survival curve and time dependent Receiver operating characteristic (ROC) curve were analyzed to assess the predictive ability of the model (Saha-Chaudhuri and Heagerty, 2013).

### 2.10 Correlation analyses of transmembrane 4 superfamily and tumor mutation burden/microsatellite instability

Spearman’s correlation analysis in the ggstatsplot package was used to determine the association between TM4SF gene expression and tumor mutation burden (TMB) and microsatellite instability (MSI) scores—two emerging biomarkers related to immunotherapy response (Zhao et al., 2019). TMB and MSI scores were got from the TCGA database. Abscissa and ordinate represent gene expression distribution and TMB/MSI fraction distribution respectively. The density curve on the right and upper represent the distribution trend of TMB/MSI score and gene expression respectively. The values of the *p*-value, correlation coefficient, and calculation method were represented on the top area.

## 3 Results

### 3.1 Expression of transmembrane 4 superfamily in papillary thyroid carcinoma

**FIGURE 1:**
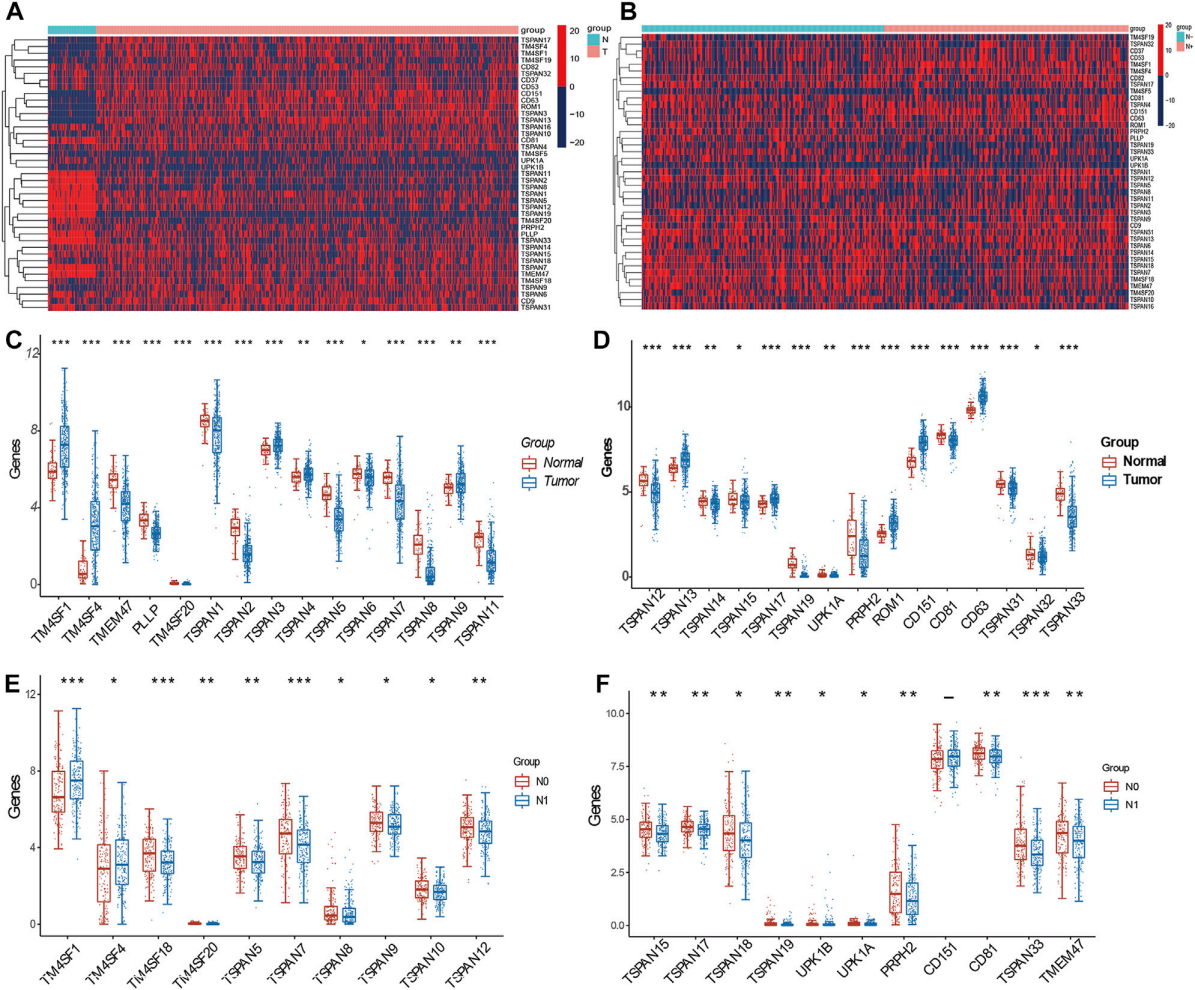
Expression of TM4SFs in thyroid papillary carcinoma. **(A)** Differentially expressed mRNA transcript heatmap between tumor and normal group of 41 TM4SF family members in PTC patients. Red represents high expression and blue represents low expression; **(B)** Differentially expressed mRNA transcript heatmap between N0 and N1 group; **(C,D)** TM4SF members with significantly different expression between tumor and normal group; and **(E,F)** between N0 and N1 group. (*p < 0.05;**p < 0.01; and ***p < 0.001).

We identified 30 DEGs among the 41 TM4SFs in PTC (Figures 1A,C,D). The expression of *TMEM47*, *PLLP*, *TM4SF20*, *TSPAN1*, *TSPAN2*, *TSPAN5*, *TSPAN6*, *TSPAN7*, *TSPAN8*, *TSPAN11*, *TSPAN12*, *TSPAN14*, *TSPAN15*, *TSPAN19*, *UPK1A*, *PRPH2*, *CD81*, *TSPAN31*, *TSPAN32*, and *TSPAN33* were downregulated in the tumors rather than upregulated in the normal. and *TM4SF1*, *TM4SF4*, *TSPAN3*, *TSPAN4*, *TSPAN9*, *TSPAN13*, *TSPAN17*, *ROM1*, *CD151*, and *CD63* were upregulated in the tumor group. We identified 21 DEGs between the N0 and N1 groups (Figures 1B,D,E). The expression of *TM4SF1*, *TM4SF4*, *UPK1B*, and *CD151* were upregulated in the N1 group and *TM4SF18*, *TM4SF20*, *TSPAN5*, *TSPAN7*, *TSPAN8*, *TSPAN9*, *TSPAN10*, *TSPAN12*, *TSPAN15*, *TSPAN17*, *TSPAN18*, *TSPAN19*, *UPK1A*, *PRPH2*, *CD81*, *TSPAN33*, and *TMEM47* were upregulated in the N0 group.

### 3.2 Differentially expressed genes in various clinicopathological stages of papillary thyroid carcinoma

**FIGURE 2:**
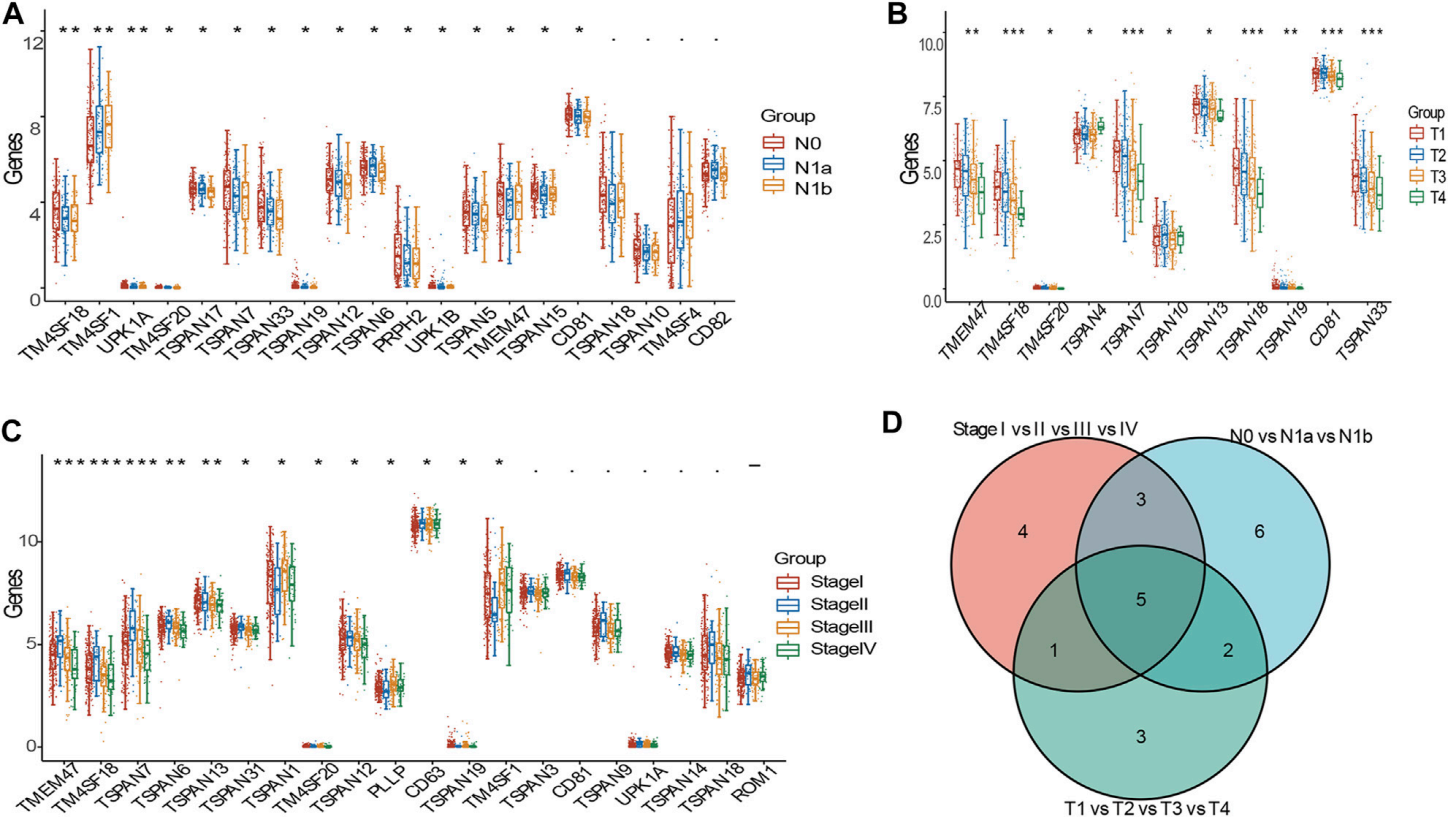
DEGs of TM4SFs in various clinicopathologic stages in patients with PTC. **(A)** TM4SF members with significantly different expression in various N stages; **(B)** and in various T stages; **(C)** and in various TNM stages; **(D)** The intersection of the three sets above of differently expressed genes. (*p < 0.05;**p < 0.01; and ***p < 0.001).

We assessed the relationship between TM4SF expression and clinicopathological stage, including various N, T, and TNM stages. In the N (N0, N1a, and N1b) stages, we identified 16 DEGs (*TM4SF1*, *TMEM47*, *TM4SF18*, *TM4SF20*, *TSPAN5*, *TSPAN6*, *TSPAN7*, *TSPAN12*, *TSPAN15*, *TSPAN17*, *TSPAN19*, *UPK1B*, *UPK1A*, *PRPH2*, *CD81*, and *TSPAN33*; Figure 2A). In the T stages (T1, T2, T3, and T4), we found 11 DEGs (*TMEM47*, *TM4SF18*, *TM4SF20*, *TSPAN4*, *TSPAN7*, *TSPAN10*, *TSPAN13*, *TSPAN18*, *TSPAN19*, *CD81*, and *TSPAN33*; Figure 2B). In the TNM stages, we identified 13 DEGs (*TM4SF1*, *TM4SF18*, *TM4SF20*, *TSPAN1*, *TSPAN6*, *TSPAN7*, *TSPAN12*, *TSPAN13*, *TSPAN19*, *CD63*, *TSPAN31*, *TMEM47*, and *PLLP*; Figure 2C). *TSPAN19*, *TMEM47*, *TSPAN7*, *TSPAN33*, and *TSPAN10* corresponded with the intersects of the three sets of DEGs (Figure 2D) and might play critical oncogenic roles in PTC progression.

### 3.3 Protein–protein interaction network, co-expression, and functional enrichment analyses of transmembrane 4 superfamily in patients with papillary thyroid carcinoma

**FIGURE 3:**
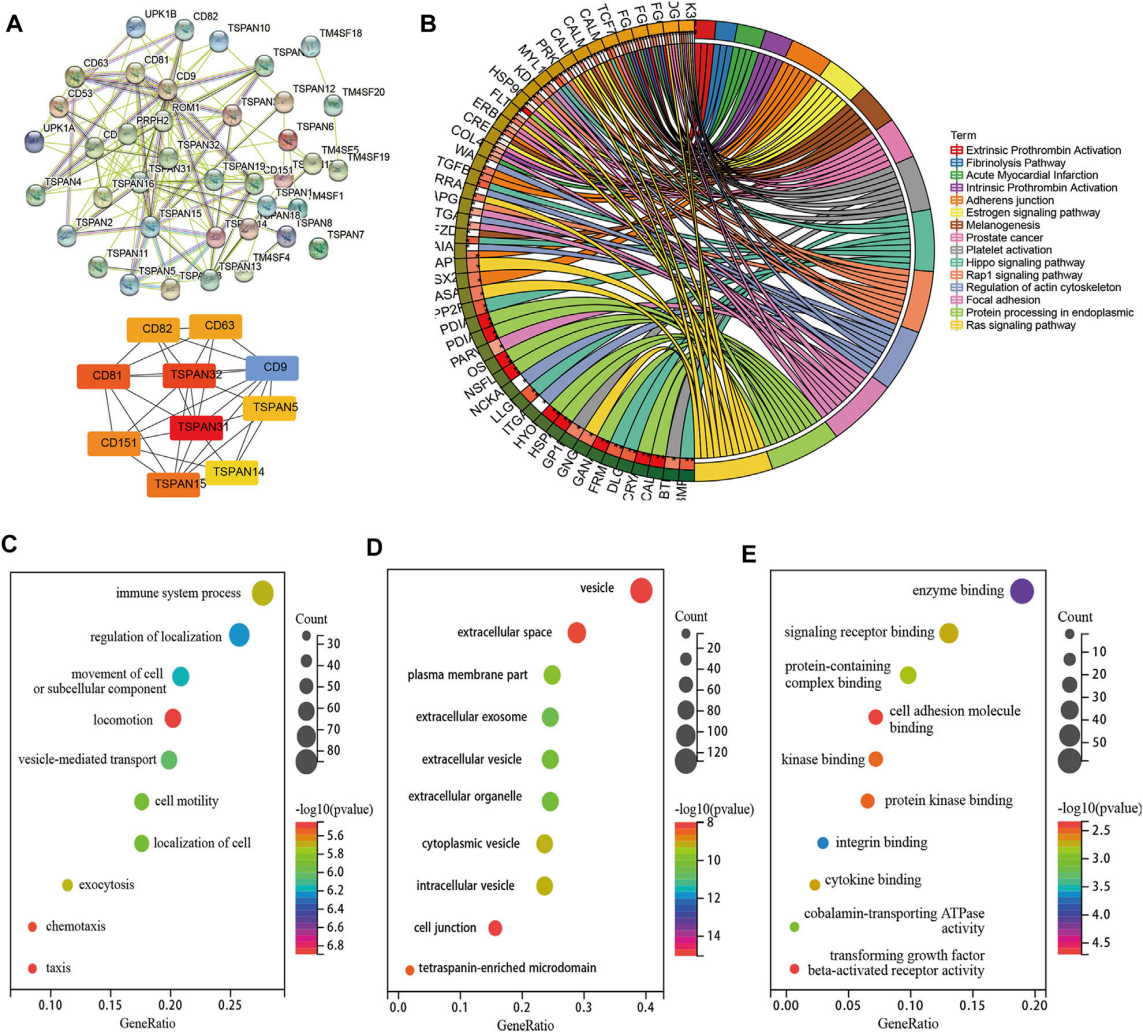
PPI Network, Co-Expression and Functional Enrichment Analyses of TM4SFs in Patients With PTC. **(A)** PPI network connectivity for proteins that co-expressed with TM4SF members *via* STRING and hub genes from the PPI network *via* cytoHubba plug-in and the MCC algorithm. KEGG **(B)** and GO [**(C)** Cellular Component; **(D)** Biological Process; **(E)** Molecular Function] enrichment analyses of those TM4SFs and co-expressed genes.

The PPI network consisted of 16 nodes and 111 edges with *p* < 0.001. The cytoHubba plug-in and the MCC algorithm were used to select the hub gene d from the PPI network as shown in Figure 3A. We performed KEGG and GO enrichment analyses of the top ten co-expressed TM4SF genes and identified 16 different KEGG signaling pathways related to LNM (*p* < 0.05) (Figure 3B), including the “integrins and other cell-surface receptors,” “Fibrinolysis Pathway,” “Focal adhesion,” “protein processing in endoplasmic reticulum,” “Hippo signaling,” and “Ras signaling” pathways. The “integrins and other cell-surface receptors” pathway was the most significantly enriched functional category. In the biological processes (BP) category of GO enrichment, several terms related to transport of substances and metabolism were identified, including immune system processes, regulation of localization, movement of cell or subcellular. The cellular components (CC) category showed that TM4SFs were most distributed in the vesicle, extracellular space, and plasma membrane (Figures 3C–E). These results suggest that TM4SFs would promote tumor progression by regulating these functions and pathways in PTC.

### 3.4 Binary logistic regression and receiver operating characteristic analyses identified *TM4SF1* as an indicator of LNM in papillary thyroid carcinoma

**FIGURE 4:**
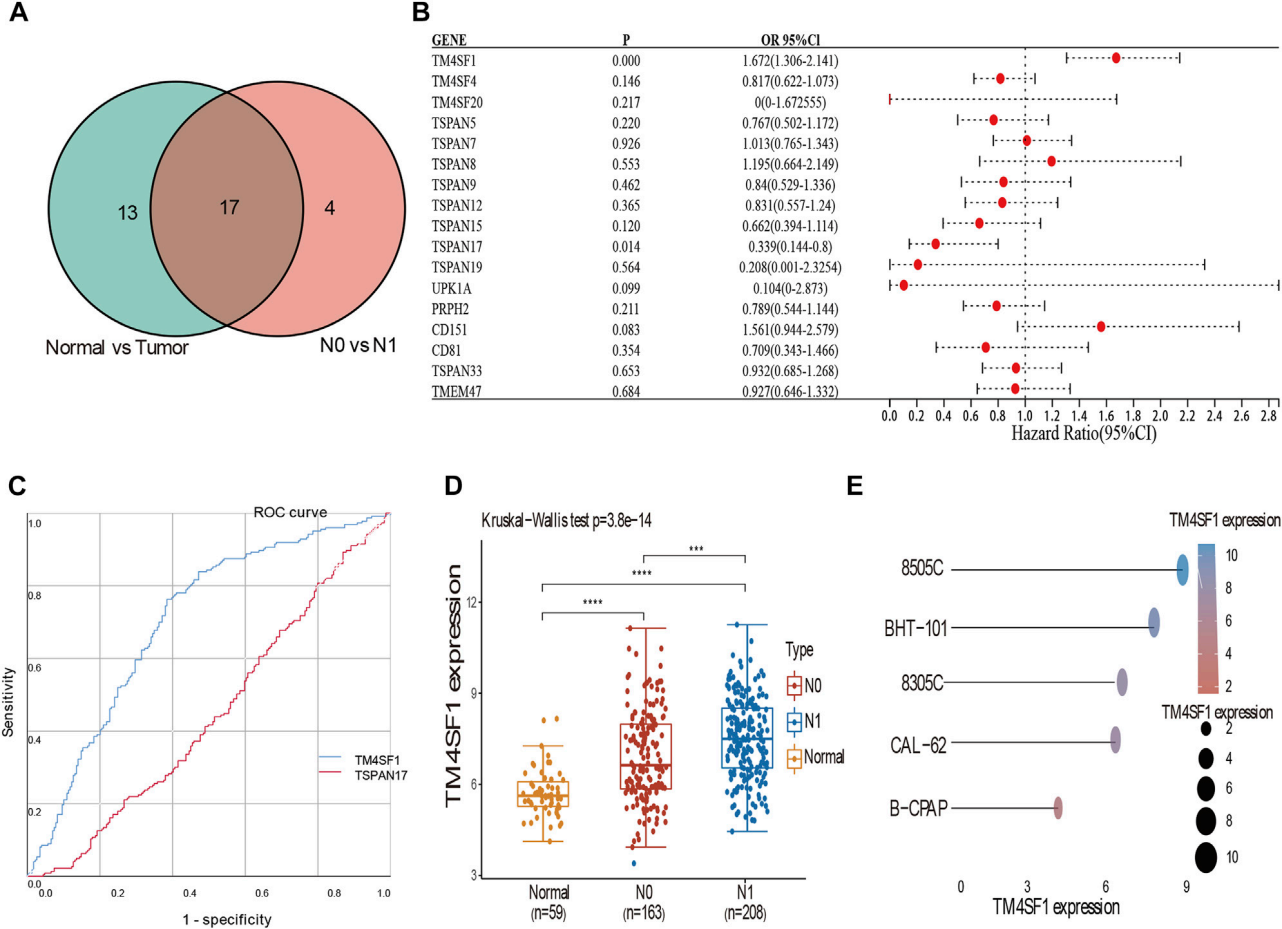
Binary logistic regression and ROC analyses suggested TM4SF1 as an indicator of LNM in PTC. **(A)** Veen diagram of the intersection of these DEGs among tumor vs. normal, N0 vs. N1; **(B)** Forest plot of data from the binary logistic regression analysis revealing genes (TSPAN17, TM4SF1) independently associated with LNM in PTC (*p* < 0.05); **(C)** ROC curve analyses of TM4SF1 (AUC = 0.702) and TSPAN17 (AUC = 0.555) to assess the diagnostic value in LNM (*p* < 0.05); **(D)** Expression of TM4SF1 mRNA in N0 vs N1 vs Normal group (*p* < 0.05); **(E)** The expression distribution of TM4SF1 mRNA in different thyroid cancer cell lines. The abscissa represents the expression distribution of mRNA and the ordinate represents different cell lines, different colors and the size of dots represent expression.

We identified 17 DEGs that corresponded between tumor vs normal and N0 vs N1 (*TM4SF1*, *TM4SF4*, *TM4SF20*, *TSPAN5*, *TSPAN7*, *TSPAN8*, *TSPAN9*,*TSPAN12*, *TSPAN15*, *TSPAN17*, *TSPAN19*, *UPK1A*, *PRPH2*, *CD151*, *CD81*, *TSPAN33*, and *TMEM47*; Figure 4A). The binary logistic regression performed with DEGs as the independent variable and LNM as dependent variable showed that *TM4SF1* and *TSPAN17* were independent factors (Figure 4B). *TM4SF1* (AUC = 0.702) was more closely associated with the occurrence of LNM compared with *TSPAN17* (AUC = 0.555, *p* < 0.01; Figure 4C). *TM4SF1* was also expressed in normal vs N0 vs N1 of the different THCA cell lines. The N1 group had the highest expression of *TM4SF1*, while the normal group had the lowest expression (Figure 4D). The expression of *TM4SF1* was higher in more malignant than in less malignant cells (Figure 4E). These findings suggest that *TM4SF1* is a potential indicator of LNM in PTC.

### 3.5 TM4SF1 facilitates epithelial–mesenchymal transition in papillary thyroid carcinoma

**FIGURE 5:**
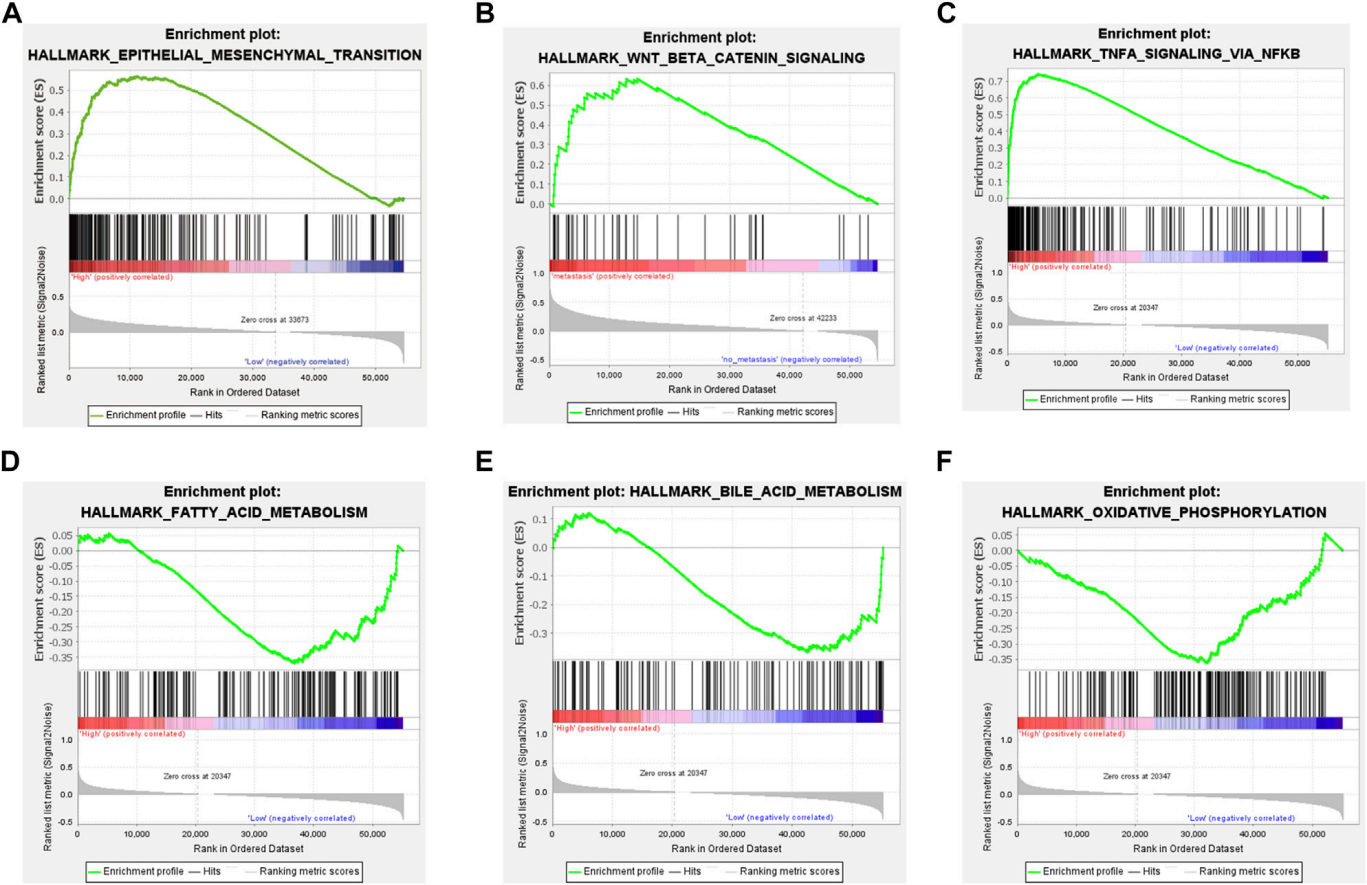
TM4SF1 facilitates epithelial-mesenchymal transition in PTC based on GSEA. **(A)** Epithelial-mesenchymal transition pathway; **(B)** Wnt/β-Catenin pathway; and **(C)** TNFA signal *via* NFKB pathway enriched in TM4SF1 high expression group; and **(D)** Fatty acid metabolism pathway; **(E)** Bile acid metabolism pathway; and **(F)** Oxidative phosphorylation pathway enriched in TM4SF1 low expression group.

To explore the underlying mechanism by which TM4SF1 promotes PTC progression and LNM, we used GSEA to identify the biological process and signaling pathway associated with *TM4SF1* expression. We found that *TM4SF1* expression was positively correlated with the upregulation of genes during epithelial–mesenchymal transition (Figure 5A)—a key event in epithelium-derived cancer cell migration and dissemination to metastatic organs. *TM4SF1* levels were associated with regulation of the Wnt/β-catenin pathway, TNFA signal *via* NFKB pathway, Fatty acid metabolism pathway, Bile acid metabolism pathway and Oxidative phosphorylation pathway in PTC (Figures 5B–E).

### 3.6 TM4SF1 overexpression was associated with DNA promoter hypomethylation

**FIGURE 6:**
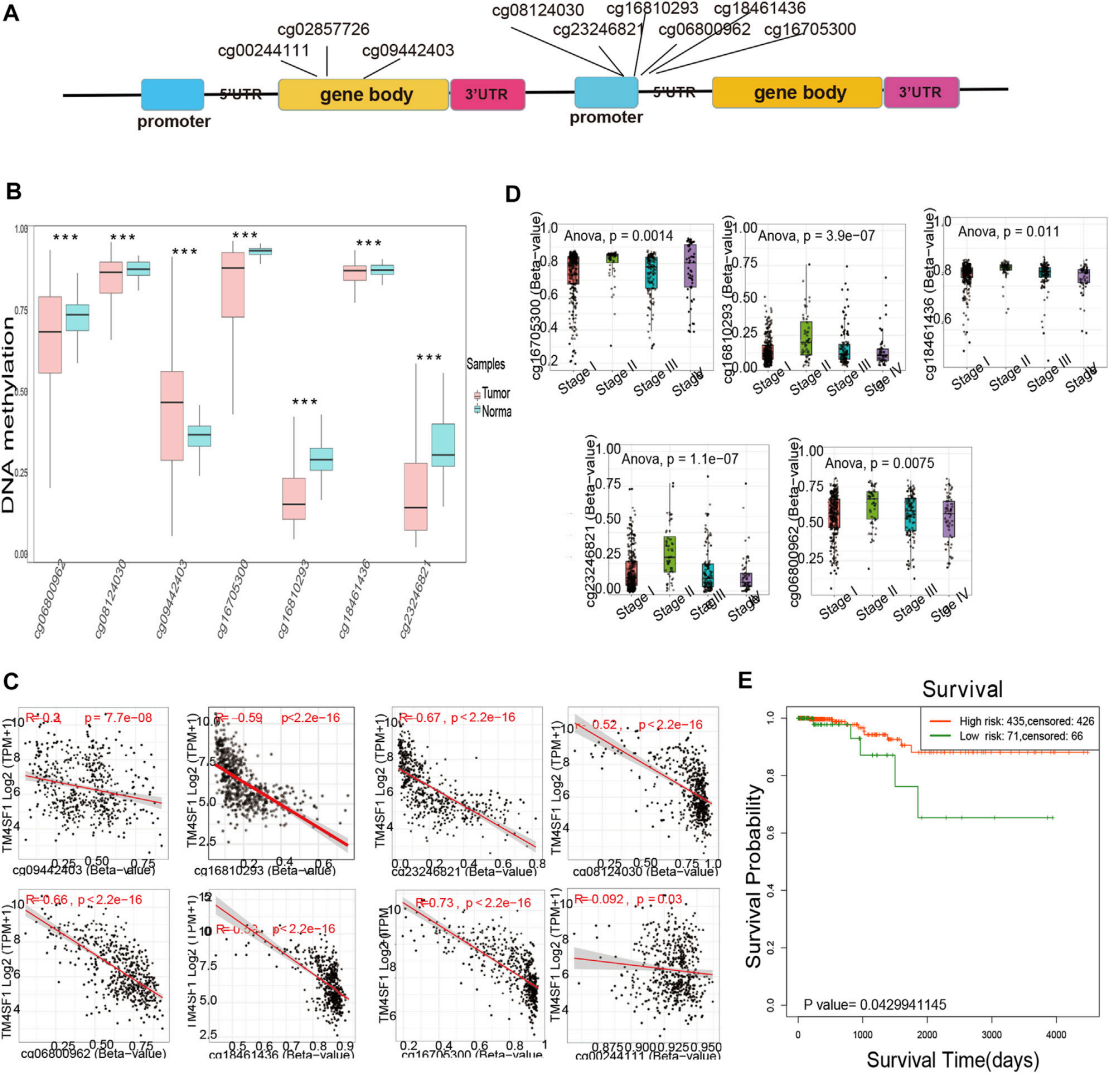
TM4SF1 overexpression was associated with DNA promoter hypomethylation in PTC. **(A)** Location of methylation probes and exons across the TM4SF1 locus; **(B)** Differentially methylated probes located across the TM4SF1 locus between normal and tumor group *via* Survival Meth database (***, *p* < 0.001); **(C)** Correlation analyses of methylation probes and TM4SF1 expression *via* SMART database; **(D)** Significantly different methylated probes of TM4SF1 in different clinical stages; **(E)** Kaplan-Meier curves for survival analysis of TM4SF1 between high-and low-risk groups.

To investigate the mechanism of *TM4SF1* upregulation in PTC, we examined the role of promoter DNA methylation. We identified 9 methylation probes (cg00244111, cg02857726, cg06800962, cg08124030, cg09442403, cg16705300, cg16810293, cg18461436, and cg23246821) in chromosome 3, of which cg23246821, cg18461436, cg16810293, cg16705300, cg09442403, cg08124030, and cg06800962 were differentially methylated (Figures 6A,B). All of the probes located on the promoter regions were associated with hypomethylation in the tumor group (Figures 6B,S.Table 1). The expression of *TM4SF1* was negatively correlated with the methylation of each probe (Figure 6C), which implied that DNA promoter hypomethylation might be the mechanism of *TM4SF1* upregulation in PTC. Further, cg23246821, cg18461436, cg16810293, cg16705300, and cg06800962 were differentially methylated in the various cancer stages (Figure 6D). Survival analysis based on *TM4SF1* methylation showed that PTC patients in the low-risk group had longer survival times. (*p* < 0.05, Figure 6E).

**TABLE 1:** Multivariable cox regression analysis for CD9, TM4SF4, TSPAN2, and TSPAN16.

Gene	HactR	*p* Value	95% Cl lower	95% Cl upper	Exp(B)
TSPAN2	2.407546477	0.001865144	1.384111839	4.18772521	0.87860817
CD9	0.379690563	0.019369755	0.168621648	0.854960945	−0.968398667
TSPAN16	265.4585087	0.097890825	0.357785128	196956.8166	5.581458552
TM4SF4	0.751836224	0.152161237	0.508823518	1.110911127	−0.285236766

### 3.7 Transmembrane 4 superfamily affect the prognosis and survival of papillary thyroid carcinoma patients *via* immune infiltration

**FIGURE 7:**
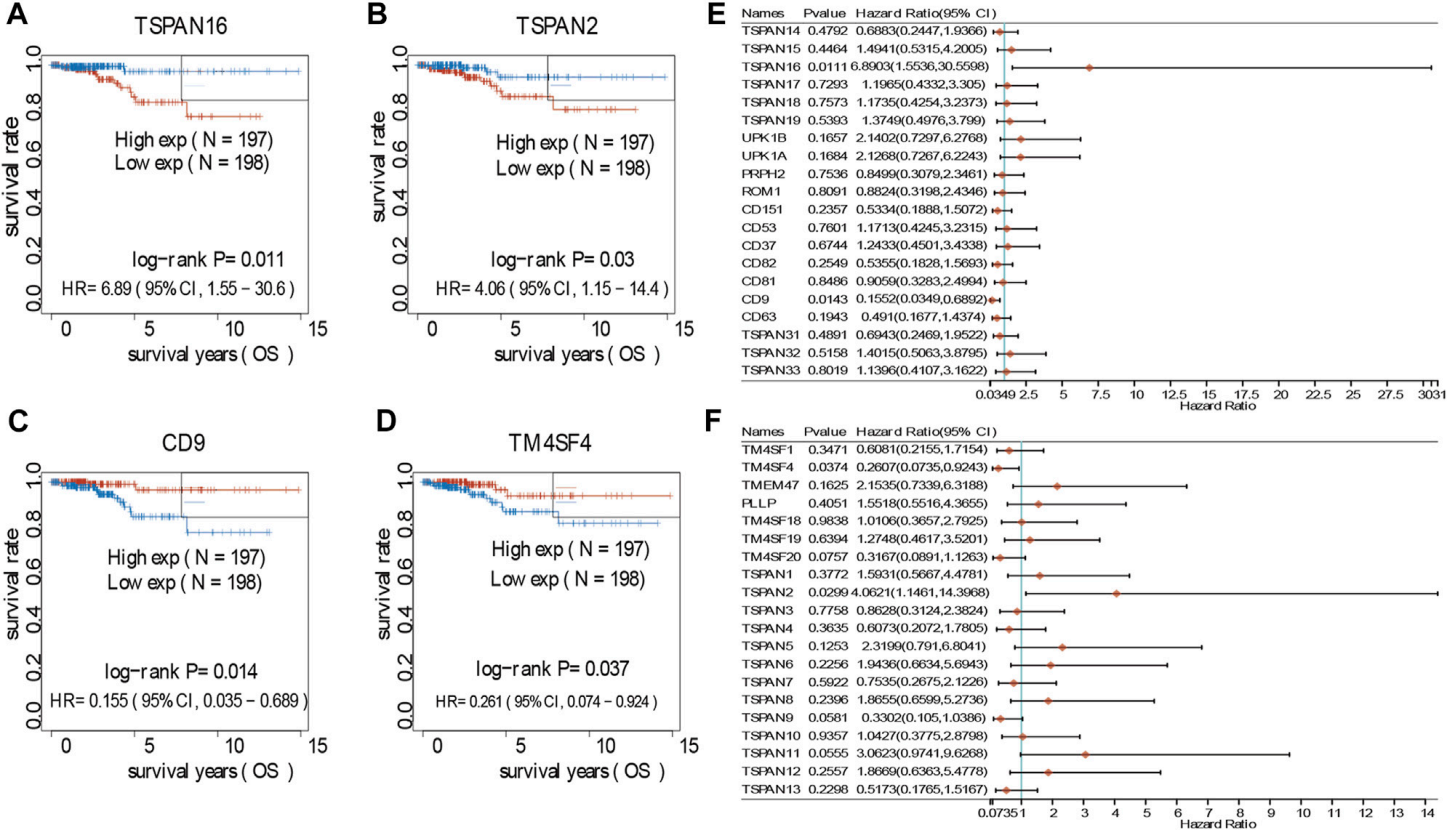
Survival analyses of TM4SFs in PTC patients. **(A–D)** Survival analyses curves of TM4SF family members with statistical significance in PTC (*p* < 0.05); **(E,F)** Forest plot of survival analysis of TM4SFs for PTC patients.

**FIGURE 8:**
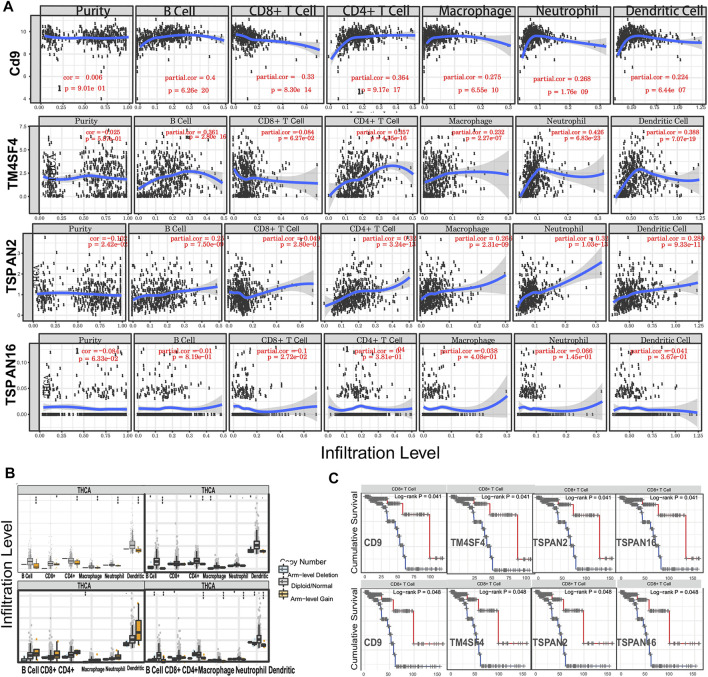
TM4SFs affect the prognosis and survival of PTC patients *via* immune infiltration. **(A)** The correlation of TM4SF members and tumor infiltrating immune cells *via* Tumor Immune Estimation Resource (TIMER); **(B)** The comparison of tumor infiltration levels of PTC with different somatic copy number alterations (SCNAs) for TM4SF members (deep deletion (−2), arm-level deletion (−1), diploid/normal (0), arm-level gain (1), and high amplification). **(C)** The 10-year and 15-year immune cell infiltration survival curve of TM4SFs in PTC patients (TIMER).

In investigating the prognostic value of TM4SFs, we identified four genes (*CD9*, *TM4SF4*, *TSPAN2*, and *TSPAN16*) associated with OS in PTC patients (Figures 7A–D). The univariate Cox proportional hazards regression results are presented in Figures 7E,F. Based on the transcriptional level of these genes in the TIMER database, we found that all four genes were involved in immune cell infiltration and tumor-related inflammatory responses. *CD9*, *TM4SF4*, and *TSPAN2* were positively related to the infiltration of B cells, neutrophils, CD4^+^ T cells, dendritic, cells and macrophages (*p* < 0.05, Figure 8A). *CD9* and *TSPAN16* were negatively related to the infiltration of CD8^+^ T cells. The SCNAs for the genes could influence immune infiltration (Figure 8B). All four genes affected the 10-year and the 15-year OS rates through the immune infiltration of CD8^+^ cells (Figures 8C,D), which provide further evidence that TM4SFs could affect the prognosis and survival of PTC patients *via* immune infiltration. Following,multivariable cox regression analysis was performed and only CD9 (*p* < 0.05) and TSPAN2(*p* < 0.01) remained prognostic factors for OS. (Table 1). As the unique indicator with *p* value less than 0.01, TSPAN2 has great potential to become a prognostic marker of PTC progression.

### 3.8 Construction of the prognostic model for papillary thyroid carcinoma

**FIGURE 9:**
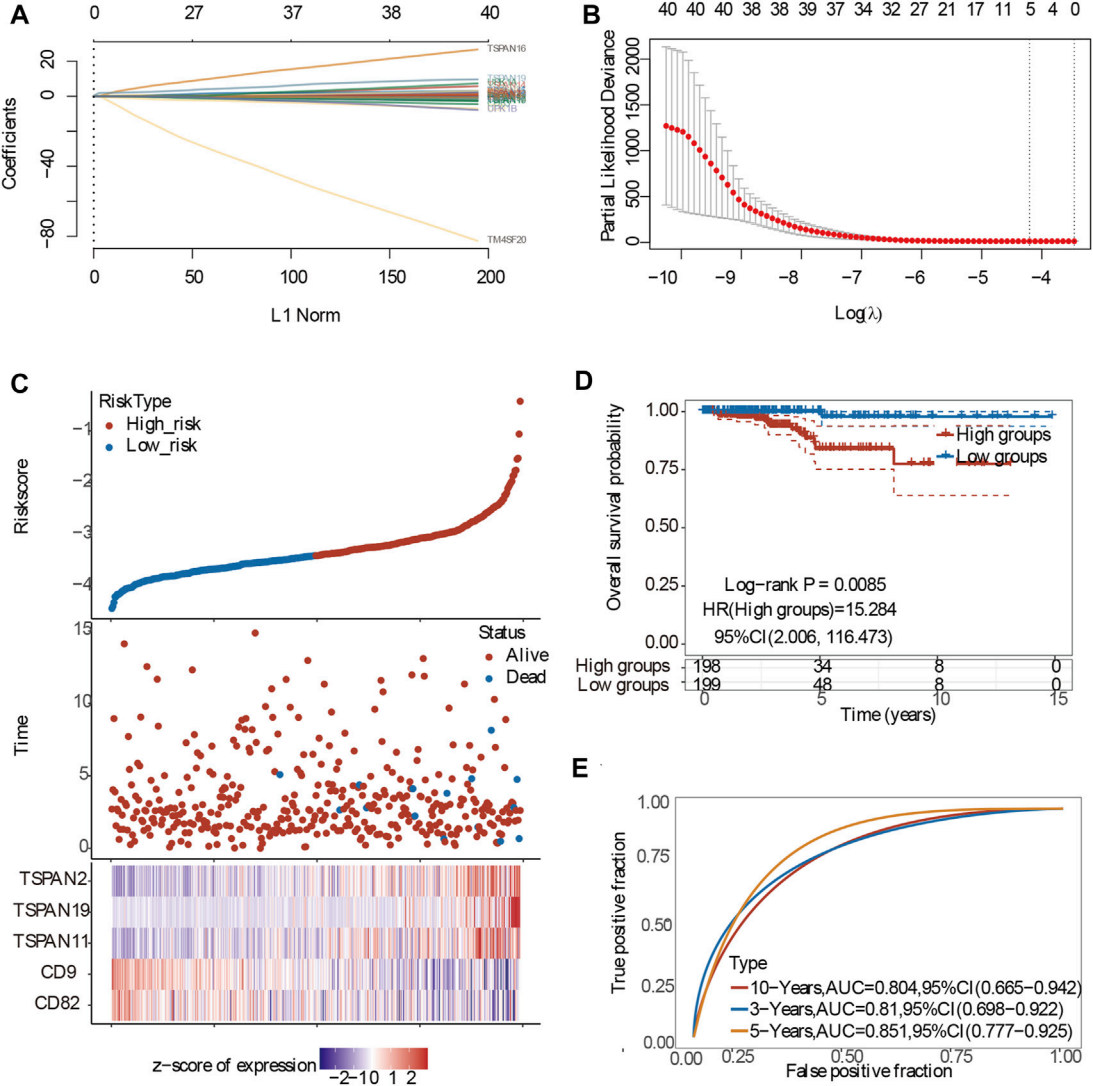
Construction of the prognostic model of PTC patients. **(A)** Processes of LASSO Cox model fitting. Each curve represents a gene. The trend of each coefficient against the L1-norm is plotted whenλchanges. L1-norm is the total absolute of non-zero coefficients. LASSO, least absolute shrinkage and selection operator; **(B)** λ selection by 10-fold cross-validation. Continuous upright lines are partial likelihood deviance ±SE; dotted lines are depicted at the optimal values by minimum criteria (lambda.min, left vertical dotted line) and 1-SE criteria (lambda.1se, right vertical dotted line). The partial likelihood deviance with changing of log (*λ*) was plotted. **(C)** The risk scores distribution, survival status, and gene expression patterns of patients in high and low-risk groups; **(D)** The risk score and best cut-off value of model; **(E)** The AUCs for 3-year,5-year and 10-year OS.

To conduct the best prognostic model, we first conducted Collinearity Diagnostics analysis with 41 TM4SF genes. The result showed that the VIF of multiple genes is greater than 5, and the VIF of CD37 and CD53 were even greater than 15 (S.Table2), which suggested that there was a multiple collinearity relationship among these 41 genes. Therefore, we adopted LASSO-penalized Cox analysis to identify hub genes and constructed the prognostic model (Figures 9A,B). On the basis of the coefficients weighted by LASSO Cox regression analysis, a prognostic model was conducted, there were five genes (CD82, CD9, TSPAN11, TSPAN19 and TSPAN2) obtained with non-zero coefficients, and the risk score was as follows: Riskscore=(-0.0058)*CD82+(-0.4994)*+(0.1584)*TSPAN11+(1.7597)*TSPAN19+(0.2694)*TSPAN2 (lambda.min = 0.0149). The risk score distribution, gene expression patterns, and survival status of patients in high-risk and low-risk groups were presented in Figures 9C,D. The AUCs for 3-year, 5-year, and 10-year OS were 0.81, 0.851, and 0.804, respectively (Figure 9E), indicating that this model had excellent predictive value.

### 3.9 Correlation between transmembrane 4 superfamily and tumor mutation burden/microsatellite instability in papillary thyroid carcinoma

**FIGURE 10:**
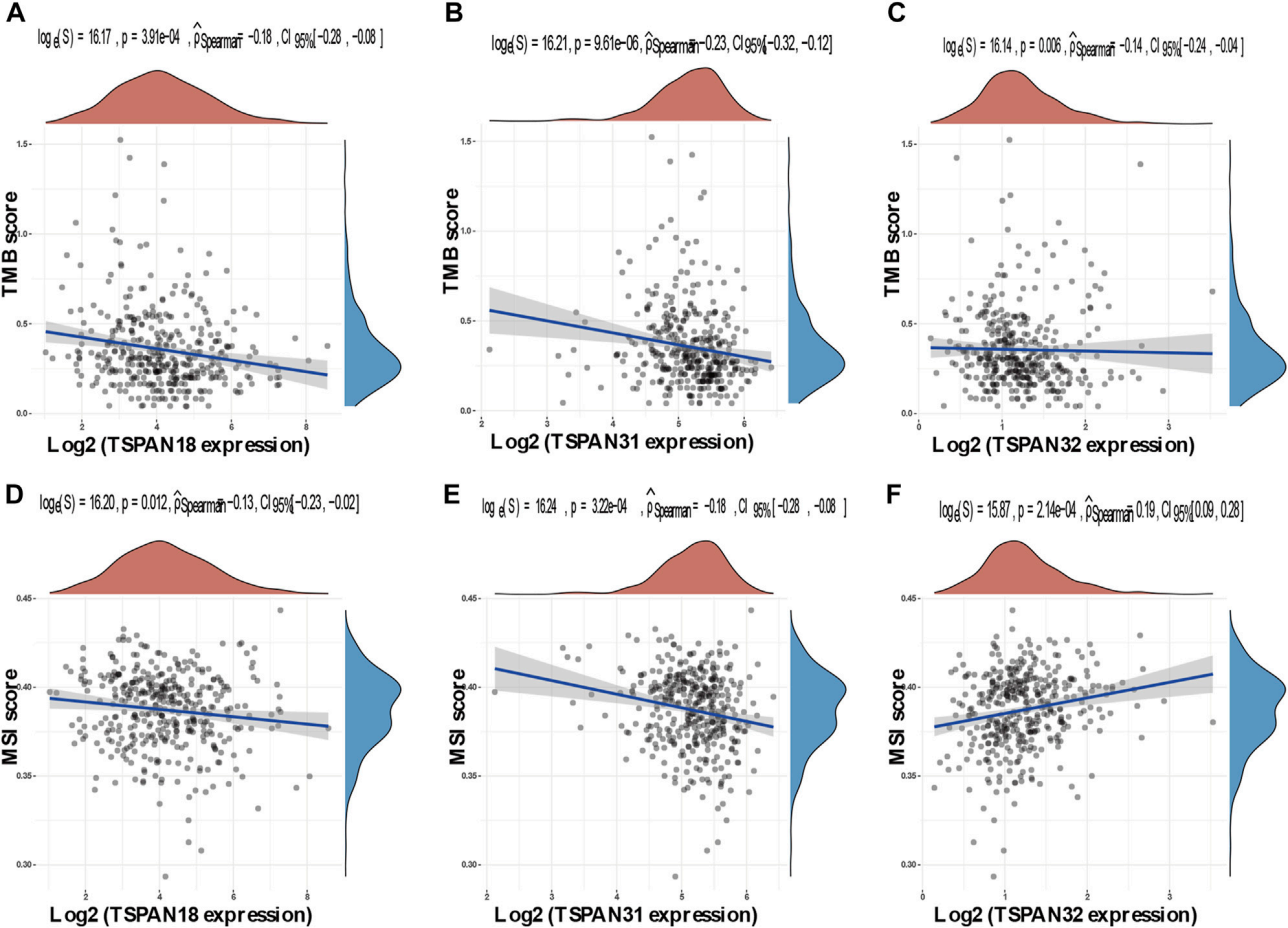
Correlation between TM4SFs and TMB/MSI in PTC patients. **(A–C)** A stick chart shows the relationship between the TM4SFs gene expression and TMB in diverse tumors. The red curve represents the correlation coefficient, and the blue value represents the range; **(D–F)** A stick chart shows the association between the TM4SFs gene expression and MSI in diverse tumors. Correlation analysis was performed using Spearman’s method.

The horizontal and the ordinate axis represented the correlation coefficient between TM4SFs and TMB or MSI, and the different types of cancer. The size and different colors of the dots represented the size of the correlation coefficient and the level of significance. Among the 41 TM4SFs, 3 members (*TSPAN18*, *TSPAN31*, and *TSPAN32*) were correlated with both TMB and MSI (Figure 10).

## 4 Discussion

Patients with differentiated THCA (papillary and follicular) have a good prognosis. The recurrence and/or metastasis seriously impact the prognosis (Van Nostrand, 2018; Fugazzola et al., 2019). Therefore, it is very important to predict and identify patients with poor prognosis as early as possible, which makes the prediction of LNM and prognosis of PTC become a critical problem.

The TM4SF protein family includes at least 16 members, most of which are leukocyte surface proteins, and the superfamily is characterized by four highly conserved transmembrane domains of cell surface proteins, making TM4SF proteins especially well adapted in tumor invasion and migration. The tetraspanins, CD151 and CD9, were previously found to interact with JAM-A and directly regulate α3β1 integrin activity to promote tumor invasion and migration (Thölmann et al., 2022). Recent evidence suggested that tetraspanins have an important impact on mitochondria turnover and regulation of cellular metabolism, which can promote metastasis (Toribio and Yáñez-Mó, 2022). The expression of several TM4SF members was dysregulated in tubal pregnancy as well as cervical cancer, hematologic malignancy, and glioma (Holters et al., 2013; Yang et al., 2016; Gao et al., 2018; Gao et al., 2022). Previous studies have linked TSPAN27 and TM4SF1 with the occurrence of PTC(Haddad et al., 2018), and TM4SF1 was reported to be a useful biomarker in the diagnosis and treatment of non-small cell lung cancer (Ma et al., 2018). In the present study, we found that *TM4SF1* is also a potential diagnostic marker of LNM and that *TSPAN2*, a TM4SF gene, is a prognostic marker of PTC progression.

Different from other TM4SF proteins, the extracellular protein ring of TM4SF1 does not contain four but only two cysteine residues. Therefore, TM4SF1 may belong to a distant branch of the TM4SF family (Boucheix and Rubinstein, 2001). In our previous study of PTC patients, we found that hyper-expression and hypomethylation of *TM4SF1* were associated with LNM (Wang et al., 2022). In the present study, the result showed that *TM4SF1* expression was an independent risk factor for LNM. The GSEA analysis suggested that *TM4SF1* could promote LNM by regulating EMT, the Wnt/β-Catenin signal pathway, and TNFα signaling *via* the NF- κB pathway. LNM involves complex biological mechanisms, of which EMT is a core step driving the metastasis cascade. During EMT, epithelial cells lose polarity and spread to the lymphatic vessels. TM4SF1 has been found to increase the degradation of the extracellular matrix by cancer cells in a variety of tumors, leading to cancer cell invasion and metastasis (Cao et al., 2018; Kim et al., 2018; Ma et al., 2018). The cytoplasmic domain of E-cadherin can bind to genes targeting the Wnt pathway, resulting in the destruction of the E-cadherin/β-catenin adhesion complex and promoting the nuclear translocation of β-catenin and transcriptional activation of the target genes (Li et al., 2022).

Abnormal DNA methylation plays a significant role in the occurrence and development of THCA (Acuña-Ruiz et al., 2022; Birden et al., 2022; Khoshfetrat et al., 2022). Demethylation and hypomethylation are known to increase gene expression and, in turn, regulate tumor formation, development, and metastasis (Kazi et al., 2015). We found that DNA hypomethylation in the promoter region determined the aberrant expression of *TM4SF1* in PTC patients. Survival analysis was conducted by integrating the contribution of clinical data and DNA methylation-related functional elements obtained from the Cox proportional hazards regression model. SurvivalMeth divided patients into two groups (high risk and low risk) based on the risk score and the maxstat model was used to evaluate the cut-off points of risk groups. Result showed that patients in higher risk group had a poorer prognosis (*p* < 0.05). However, the mechanism of how abnormal DNA methylation of TM4SF1 affects prognosis remains to be further studied.

We identified four genes (*CD9*, *TM4SF4*, *TSPAN2*, and *TSPAN16*) associated with OS in PTC patients. Because of GO and KEGG analyses of TM4SF genes showed that these genes may promote LNM of PTC by regulating pathways including “integrins and other cell-surface receptors,” “Fibrinolysis Pathway,” “Focal adhesion,” and functions including “immune system processes,” “regulation of localization,” “movement of cell or subcellular,” “extracellular space”, which suggested that TM4SF genes probably be closely related to tumor microenvironment (TME) and immune function, we speculated that these four genes may affect the prognosis by regulating TME and immune function. Coincidentally, our immune analysis results from TIMER database are consistent with our assumptions. Results showed that all of these four genes participated in immune cell infiltration and tumor-related inflammatory responses. TME is a non transforming region around tumor cells, mainly composed of cancer associated fibroblasts (CAF) and immune cells. Tumor-associated macrophages (TAM) are the most common component among infiltrating immune cells. Research revealed that macrophage infiltration rate was significantly increased in PTC compared to in benign tumors and was correlated with lymph node metastasis and poor prognosis (Qing et al., 2012; Kim et al., 2013; Fang et al., 2014; Jung et al., 2015). The density of TAM is related to the invasion and metastasis of PTC, which is the result of the combination of CXCL8 secreted by TAM and CXCR1/2 secreted by PTC(Fang et al., 2014). In addition, 95% of PTC showed mast cells, and the amount of TMC was related to the expansion of extrathyroid tumors (Melillo et al., 2010). This is because on the one hand, it could secrete histamine, CXCL1/GRO-α and CXCL10/IP-10 to promote the proliferation of thyroid cancer cells (Petty and Yang, 2017), and on the other hand, it could initiate EMT by secreting TNF, IL-6 and CXCL8/IL-8, which were essential in EMT process (Visciano et al., 2015). EMT-activated cancer cells can make use of immune-checkpoint molecules to achieve immune escape by transmitting “don’t eat me” signals to macrophages to survive and metastasize (Mohme et al., 2017; Noman et al., 2018).

The NCCN thyroid cancer clinical practice guidelines suggest that PD-1/PD-L1 inhibitor can be used to treat THCAs with high tumor mutation load (Haddad et al., 2018). Cytotoxic T lymphocyte-associated antigen 4 and PD-1/PD-L1 inhibitors are particularly common immune checkpoint inhibitors in clinical research. Their main mechanism of action is the reactivation of T lymphocytes that specifically kill tumor cells. These inhibitors have had some success in the treatment of non-small cell lung cancer, melanoma, bladder cancer, renal cell carcinoma, head and neck carcinoma, colon cancer, among other malignant tumors (Grecea et al., 2021). In this regard, we investigated the association between TMB/MSI and TM4SFs to screen PTC patients that might be sensitive to immune checkpoint inhibitors. In the present study, we found that high expression of *TSPAN18*, *TSPAN31*, and *TSPAN32* was associated with both TMB and MSI in PTC patients, suggesting that these three genes are makers of advanced PTC and potential targets for immunotherapy.

This is the first study to systematically assess the expression and potential mechanisms of TM4SFs in PTC. We identified *TSPAN2* as a prognostic marker of PTC occurrence and TM4SF1 as a diagnostic of LNM in PTC. We propose that TM4SFs are important biomarkers in the diagnosis of LNM and could contribute to the prediction of effective treatments and long-term prognosis in PTC. Our study provides a potential alternative to prophylactic neck dissection in determining LNM that could improve quality of life (by avoiding redundant surgeries) and survival rate (by providing better prognostic markers). We acknowledge some limitations to our study. Most of our results are based on bioinformatic analysis. Although we confirmed the differential expression and methylation of TM4SF1 between PTC patients with LNM, further studies are required to test its diagnostic efficacy and its mechanisms of action require experimental validation. Our research group intends on implementing and expanding these findings in future *in vivo*, *in vitro*, and clinical studies.

## Data Availability

The original contributions presented in the study are included in the article/Supplementary Material, further inquiries can be directed to the corresponding authors.
