# Supplementary material for: Potential diagnostic of lymph node metastasis and prognostic values of TM4SFs in papillary thyroid carcinoma patients
Source: Front Cell Dev Biol. 2022 Dec 8;10:1001954. doi: 10.3389/fcell.2022.1001954 (PMC9773885; doi:10.3389/fcell.2022.1001954)
Supplement: Supplementary file 2 [file Table1.docx]

**Differentially methylated probes located across the TM4SF1 locus**

| Probe Id | Tumor | Normal  samples | Delta value | Fold-change | P_value |
| --- | --- | --- | --- | --- | --- |
| cg23246821 | 0.205992 | 0.328568 | -0.12257 | 0.626938 | 3.00127E-13 |
| cg18461436 | 0.851005 | 0.870587 | -0.01958 | 0.977507 | 8.10256E-06 |
| cg16810293 | 0.189735 | 0.301246 | -0.11151 | 0.629833 | 1.83697E-18 |
| cg16705300 | 0.805191 | 0.92845 | -0.12325 | 0.867242 | 1.90295E-48 |
| cg09442403 | 0.436872 | 0.365563 | 0.071308 | 1.195065 | 2.21188E-09 |
| cg08124030 | 0.814749 | 0.870999 | -0.05625 | 0.935418 | 1.63934E-12 |
| cg06800962 | 0.652161 | 0.724857 | -0.07269 | 0.899709 | 1.40323E-08 |
| cg00244111 | 0.931382 | 0.93355 | -0.00216 | 0.997677 | 0.251305976 |
| cg02857726 | 0.869184 | 0.867031 | 0.002153 | 1.002483 | 0.727594334 |
