# Supplementary material for: Potential diagnostic of lymph node metastasis and prognostic values of TM4SFs in papillary thyroid carcinoma patients
Source: Front Cell Dev Biol. 2022 Dec 8;10:1001954. doi: 10.3389/fcell.2022.1001954 (PMC9773885; doi:10.3389/fcell.2022.1001954)
Supplement: Supplementary file 6 [file Table2.docx]

**Supplementary Table2.Collinearity diagnostics for 41 TM4SFs.**

| **Coefficients**a | | | | | | | |
| --- | --- | --- | --- | --- | --- | --- | --- |
| model | Unstandardized Coefficients | | Standardized Coefficients | t | Sig | Collinearity Statistics | |
|  | B | SE | Beta |  |  | Tolerance | VIF |
| (Constant) | 1.947 | 5.516 |  | 0.353 | 0.724 |  |  |
| CD151 | 0.619 | 0.393 | 0.144 | 1.576 | 0.116 | 0.297 | 3.367 |
| CD37 | 0.839 | 0.505 | 0.324 | 1.662 | 0.097 | 0.065 | 15.384 |
| CD53 | -0.707 | 0.455 | -0.309 | -1.553 | 0.121 | 0.062 | 16.006 |
| CD63 | -0.141 | 0.57 | -0.023 | -0.247 | 0.805 | 0.279 | 3.58 |
| CD81 | 0.716 | 0.695 | 0.112 | 1.03 | 0.304 | 0.207 | 4.826 |
| CD82 | 0.267 | 0.292 | 0.069 | 0.915 | 0.361 | 0.43 | 2.324 |
| CD9 | -0.483 | 0.496 | -0.102 | -0.975 | 0.33 | 0.228 | 4.395 |
| PLLP | 0.294 | 0.412 | 0.05 | 0.715 | 0.475 | 0.504 | 1.984 |
| PRPH2 | 0.176 | 0.155 | 0.067 | 1.136 | 0.257 | 0.71 | 1.408 |
| ROM1 | 0.057 | 0.503 | 0.012 | 0.113 | 0.91 | 0.23 | 4.342 |
| TM4SF1 | 0.008 | 0.176 | 0.004 | 0.047 | 0.962 | 0.3 | 3.329 |
| TM4SF18 | -0.057 | 0.343 | -0.02 | -0.166 | 0.868 | 0.17 | 5.877 |
| TM4SF19 | 0.574 | 0.426 | 0.081 | 1.346 | 0.179 | 0.69 | 1.449 |
| TM4SF20 | -4.523 | 4.042 | -0.063 | -1.119 | 0.264 | 0.791 | 1.265 |
| TM4SF4 | -0.128 | 0.136 | -0.08 | -0.945 | 0.345 | 0.347 | 2.881 |
| TMEM47 | -0.014 | 0.268 | -0.005 | -0.053 | 0.958 | 0.249 | 4.017 |
| TSPAN1 | 0.021 | 0.169 | 0.01 | 0.122 | 0.903 | 0.362 | 2.766 |
| TSPAN10 | -0.613 | 0.355 | -0.12 | -1.727 | 0.085 | 0.507 | 1.972 |
| TSPAN11 | -0.363 | 0.291 | -0.102 | -1.247 | 0.213 | 0.366 | 2.736 |
| TSPAN12 | 0.373 | 0.29 | 0.119 | 1.287 | 0.199 | 0.291 | 3.441 |
| TSPAN13 | 0.045 | 0.355 | 0.01 | 0.127 | 0.899 | 0.362 | 2.762 |
| TSPAN14 | 0.509 | 0.664 | 0.082 | 0.766 | 0.444 | 0.213 | 4.7 |
| TSPAN15 | 0.143 | 0.371 | 0.031 | 0.385 | 0.7 | 0.382 | 2.615 |
| TSPAN16 | -0.823 | 2.478 | -0.018 | -0.332 | 0.74 | 0.845 | 1.184 |
| TSPAN17 | -0.424 | 0.588 | -0.061 | -0.721 | 0.472 | 0.341 | 2.929 |
| TSPAN18 | -0.208 | 0.187 | -0.096 | -1.114 | 0.266 | 0.335 | 2.985 |
| TSPAN19 | -0.637 | 1.221 | -0.037 | -0.522 | 0.602 | 0.498 | 2.009 |
| TSPAN2 | 0.194 | 0.3 | 0.049 | 0.647 | 0.518 | 0.426 | 2.349 |
| TSPAN3 | 0.059 | 0.657 | 0.011 | 0.09 | 0.928 | 0.168 | 5.965 |
| TSPAN31 | -0.201 | 0.706 | -0.038 | -0.285 | 0.776 | 0.137 | 7.276 |
| TSPAN32 | -0.848 | 0.405 | -0.146 | -2.096 | 0.037 | 0.51 | 1.96 |
| TSPAN33 | -0.155 | 0.241 | -0.056 | -0.644 | 0.52 | 0.322 | 3.105 |
| TSPAN4 | -1.282 | 0.5 | -0.236 | -2.564 | 0.011 | 0.292 | 3.424 |
| TSPAN5 | -0.555 | 0.27 | -0.168 | -2.058 | 0.04 | 0.37 | 2.7 |
| TSPAN6 | 0.3 | 0.398 | 0.066 | 0.754 | 0.451 | 0.318 | 3.147 |
| TSPAN7 | -0.273 | 0.207 | -0.125 | -1.317 | 0.189 | 0.275 | 3.632 |
| TSPAN8 | 0.029 | 0.24 | 0.008 | 0.121 | 0.904 | 0.52 | 1.923 |
| TSPAN9 | 0.956 | 0.361 | 0.254 | 2.648 | 0.008 | 0.269 | 3.714 |
| UPK1A | 0.618 | 0.855 | 0.041 | 0.722 | 0.471 | 0.758 | 1.32 |
| UPK1B | -0.76 | 0.533 | -0.085 | -1.426 | 0.155 | 0.697 | 1.435 |
| a. Dependent variable：times | | | | | | | |

| **Supplementary Table 3.Co-expression genes of TM4SFs.** | | | | | | | | | | |
| --- | --- | --- | --- | --- | --- | --- | --- | --- | --- | --- |
| TM4SF1 | TM4SF1-AS1 | RP11-278L15.2 | TM4SF4 | ANXA2 | NBL1 | TNFRSF21 | SPOCK2 | ACTBL2 | MACC1 | F2RL1 |
| TM4SF4 | RP11-278L15.6 | TM4SF1 | RP11-278L15.2 | BMP4 | TM4SF1-AS1 | ADGRL3 | TGM1 | CFTR | RP11-54A9.1 | RAET1E |
| TM4SF5 | FGB | FGG | RP11-63P12.7 | CT47A7 | SNORA67 | RP1-288L1.4 | FGA | GC | CPN1 | CFHR2 |
| TM4SF18 | KDR | MYCT1 | CALCRL | AFAP1L1 | FLT1 | LDB2 | CDH5 | EMCN | ROBO4 | ADGRL4 |
| TM4SF19 | AC004988.1 | HTRA4 | TM4SF19-TCTEX1D2 | CHIT1 | ATP6V0D2 | GM2A | MEP1A | FGR | CYP27A1 | ITGAX |
| TM4SF20 | AC008906.1 | OR5K4 | WFDC11 | RP11-167N24.5 | ZKSCAN7P1 | RNA5SP155 | SNX18P2 | RP11-826N14.4 | USP9YP2 | RP11-462P6.1 |
| TSPAN1 | TMPRSS13 | CPAMD8 | UBAP2 | LINC01127 | TNS4 | GRK5 | F2RL1 | CNTN6 | AC002066.1 | RP11-58O9.2 |
| TSPAN2 | SEMA3C | FRMD6 | DSEL | FBXO17 | RAI14 | FBLIM1 | TNFSF10 | TPM4 | FEZ1 | PXDN |
| TSPAN3 | OAZ2 | SPINT2 | TMEM59 | HDAC1 | MXRA7 | MRFAP1L1 | OCIAD1 | MOAP1 | VWA9 | TMEM57 |
| TSPAN4 | SCAP | CDC37 | TMEM259 | SCAMP4 | RALY | CTDSP1 | NELFB | LTBP4 | MAF1 | HSF1 |
| TSPAN5 | RP11-1299A16.3 | SMAD9 | CABLES1 | ABCC1 | LOXL4 | RP13-507P19.2 | SOGA1 | KAZN | FOXE1 | ARHGAP28 |
| TSPAN6 | TOP2B | GLG1 | ERBB2 | VANGL2 | KHDRBS1 | HNRNPA0 | C7orf60 | RBM15B | PHF10 | ZSCAN2 |
| TSPAN7 | SNPH | SUOX | MTCH1 | METTL20 | PRDM11 | MLEC | COPZ1 | VDAC3 | TSR2 | PEX11A |
| TSPAN8 | CCL15-CCL14 | CCL15 | KCNG2 | LINC00475 | ATP10B | RP11-304L19.1 | HPDL | RP11-1C1.6 | RP11-304L19.3 | RP11-61L23.2 |
| TSPAN9 | SLIT3 | CPE | FUT11 | CAB39 | SCAP | SEMA3F | CADM1 | TSC22D1 | SCN2B | TSPYL4 |
| TSPAN10 | ZNF599 | ZNF446 | TMEM42 | WBP1 | SDR39U1 | LRRC27 | C14orf79 | MKS1 | RP11-345P4.9 | CDC37 |
| TSPAN11 | GPR68 | PITX2 | PODNL1 | RP11-119J18.1 | PGLYRP3 | XAGE1B | XAGE1A | SFTPA2 | TTC39C | SPTB |
| TSPAN12 | LRRC1 | ABCC1 | UBAP2 | SSFA2 | GALNT18 | MAP4K3 | POFUT1 | RRAS2 | PRKCI | NCS1 |
| TSPAN13 | CMTM6 | TMEM30A | YPEL5 | RNF103 | PDCL | ARCN1 | CASD1 | ELAVL1 | C1GALT1C1 | MOB4 |
| TSPAN14 | MEF2A | NKIRAS2 | RAPGEF1 | PDCL | ABHD17B | LRCH1 | ARHGAP21 | ATF7 | TGFBR2 | NOTCH1 |
| TSPAN15 | GNG11 | ACKR3 | TCF7L1 | SLC16A13 | FSCN1 | RP11-61L23.2 | COL4A2 | CSPG4 | TCAF2 | EEF2KMT |
| TSPAN16 | RP11-876N24.7 | RP11-159F24.2 | WDR83 | CTD-2132N18.4 | SPTBN4 | GAMT | CTD-2002J20.1 | VPS16 | EBF4 | GP1BB |
| TSPAN17 | PCNXL3 | SLC35A2 | DPP3 | ACTR1A | SURF4 | TMEM184B | YKT6 | TRPC4AP | EXT2 | KLC2 |
| TSPAN18 | FAM167A | HYOU1 | PDIA6 | JAZF1 | SORBS2 | GNA14 | PDIA4 | FHL1 | CALR | HSPA5 |
| TSPAN19 | LRRIQ1 | LRRC9 | RP11-308K2.1 | RPS15AP7 | AC000370.2 | AC013401.2 | RPL7P41 | CCDC150P1 | SLC6A15 | RP11-375I20.6 |
| UPK1B | CALML5 | RP11-1195F20.7 | TSPY1 | AC006987.6 | CALML3 | RP11-479F13.1 | CAPNS2 | KRT16 | RP11-116G8.5 | LYPD3 |
| UPK1A | RNA5SP101 | Y_RNA | MIR765 | TTTY2B | KRTAP4-16P | RP11-96J15.1 | TTTY2 | RP11-429B14.3 | RNU4-51P | RNU1-6P |
| PRPH2 | CYB561 | PDIA6 | HSPA5 | HYOU1 | GNA14 | MLEC | RP11-165E7.1 | HSP90B1 | CALR | MAGT1 |
| ROM1 | SSBP4 | PQBP1 | GPR108 | CERS5 | EML3 | CCDC12 | MAP3K11 | ADD1 | EFEMP2 | B3GAT3 |
| CD151 | SH3GL1 | SNAPC2 | RAB34 | MXRA8 | TMEM256-PLSCR3 | MYL12B | PNPLA2 | LSR | BAIAP2 | TAX1BP3 |
| CD53 | DOCK2 | HCLS1 | WAS | BIN2 | BTK | LAPTM5 | ARHGAP30 | NCKAP1L | PARVG | SASH3 |
| CD37 | SASH3 | SP140 | CD48 | BTK | SCIMP | ARHGAP30 | IRF8 | IKZF1 | IL16 | WAS |
| CD82 | LLGL1 | SERTAD2 | ANKH | ARMCX3 | PRRG1 | SOWAHA | ABHD2 | TMEM159 | RAB11FIP4 | SSX2IP |
| CD81 | YIPF3 | CREB3 | OS9 | SCAMP4 | OTUB1 | AAMP | SDF4 | USP11 | GPKOW | CDIPT |
| CD9 | CALM2 | MEAF6 | HMGB1 | LAPTM4A | LMBRD1 | RANBP3 | LCA5 | NSFL1C | ISOC1 | TOP2B |
| CD63 | NPC2 | CHMP2A | TAF10 | RNF181 | PSMB1 | KRTCAP2 | C19orf53 | TMEM219 | BCL7C | TSTA3 |
| TSPAN31 | TMBIM6 | GANAB | ATXN7L3B | DCAF11 | SERINC1 | TMEM248 | POLR2C | C6orf89 | KAT7 | RUNDC1 |
| TSPAN32 | C11orf21 | ARHGAP4 | RASAL3 | DEF6 | ZAP70 | PARVG | PSTPIP1 | LINC00528 | S1PR4 | GPSM3 |
| TSPAN33 | RNF157 | MRPS33 | RP11-715H19.2 | PPP2R2B | PPIF | KLK15 | MAPK4 | NPY4R | AC002467.7 | AIMP2 |
| TMEM47 | ITGA1 | FZD4 | FLT1 | KDR | MYCT1 | CDH5 | C4orf32 | PDGFD | LDB2 | PIK3R3 |
| PLLP | RP3-332B22.1 | PPIF | RP11-12A2.3 | DUSP15 | HOXA13 | TRIM50 | ACAT1 | KCNJ1 | CRYAB | DLG2 |
